# Case Report: Successful treatment of steroid-refractory graft-vs.-host disease following bilateral lung transplantation

**DOI:** 10.3389/frtra.2025.1682433

**Published:** 2025-10-13

**Authors:** Benjamin J. McCormick, Victoria Rusanov, Zhuo Tao, Thomas Kaleekal, Keith Wille, Manuel R. Espinoza-Gutarra

**Affiliations:** ^1^ Division of Hematology and Oncology, The University of Alabama, Birmingham, AL, United States; ^2^ Division of Pulmonary Allergy and Critical Care Medicine, The University of Alabama, Birmingham, AL, United States; ^3^ Department of Surgery, The University of Alabama, Birmingham, AL, United States

**Keywords:** graft vs. host desease, lung transplant, transplant related complications, solid organ transplant (SOP), immunosuppression

## Abstract

Graft-vs.-host disease (GVHD) is a rare but potentially fatal complication following solid organ transplantation (SOT), with limited reported cases and high mortality rates after lung transplantation. We present a case of steroid-refractory GVHD (SR-GVHD) following bilateral lung transplantation and review the literature on GVHD in SOT. A patient developed SR-GVHD affecting the skin, gut, liver, and bone marrow following bilateral lung transplantation. Initial treatment with high-dose corticosteroids was ineffective. Subsequent therapy with rabbit anti-thymocyte globulin (rATG) and ruxolitinib led to complete remission over two months. Short tandem repeat (STR) analysis aided in diagnosis and monitoring. This case highlights the importance of early diagnosis and aggressive treatment of GVHD following SOT. We propose a treatment algorithm including rapid escalation to multi-agent immunosuppression for SR-GVHD. Interdisciplinary collaboration between solid organ and stem cell transplant specialists is crucial. Further research is needed to identify optimal strategies for prevention and treatment of GVHD in SOT recipients.

## Introduction

Acute graft-vs.-host disease (GVHD) is a common complication of allogeneic hematopoietic stem cell transplantation (HSCT); however, it is rare and associated with high mortality following solid organ transplantation (SOT). There have been only 15 reported cases of GVHD following lung transplantation with greater than 80% mortality (1, 2).

GVHD typically develops 2–12 weeks after SOT with recipient skin, mucosa, gastrointestinal tract, liver and bone marrow affected. Clinical presentations include rash, nausea, anorexia, diarrhea, mucositis, fever, elevated transaminases, hyperbilirubinemia, and cytopenias. The most common causes of death from GVHD following SOT are infection and hemorrhage. The diagnosis of GVHD following SOT is made clinically and confirmed with histopathology of tissue in affected organs. Following stem cell transplantation, peripheral blood short tandem repeats (STRs) analysis is performed routinely to monitor for donor cell engraftment by quantifying the respective proportions of donor and recipient cells in the blood (3). STR analysis can also be used to monitor circulating donor derived CD3T-cells following SOT which may be associated with increased risk for GVHD (4).

We present the case of a patient with bilateral lung transplantation who developed steroid-refractory GVHD (SR-GVHD) and is now in complete remission following an extended course of immunosuppression.

## Case presentation

A 63-year-old male underwent bilateral lung transplantation for pulmonary fibrosis due to COVID-19 infection. Basiliximab was used perioperatively for immunosuppression induction. Subsequently, tacrolimus (target, 8–12 mg/dl) and mycophenolate mofetil were implemented. His initial hospital course following transplantation was uncomplicated other than diarrhea (grade 3), which had improved prior to discharge and was attributed to medications. On Day 25, he presented with stage 1 skin aGVHD with maculopapular rash which progressed to stage 2 by Day 27 affecting 50% body surface area and grade 3 gut aGVHD. On day 28, a skin biopsy confirmed grade 2 GVHD, and colonoscopy with biopsy confirmed gut GVHD on day 30. The patient was given methylprednisolone 1 mg/kg twice daily, oral budesonide 3 mg three times daily (TID), and topical 1% hydrocortisone cream TID. Chimerism analysis was performed using short tandem repeat (STR) PCR for CD3 cell lineage in peripheral blood, which confirmed 81% donor chimerism at the time of aGVHD treatment initiation.

He had improvement in cutaneous symptoms; however, he developed worsening diarrhea, cytopenias and grade 3 liver GVHD (total bilirubin, 7.3 mg/dl). Infectious workup for diarrhea was negative. Methylprednisolone was increased to 2 mg/kg daily. Ursodiol 300 mg TID was initiated. Rabbit anti-thymocyte globulin (rATG) 1.5 mg/kg for 4 days was started on Day 36 at which time methylprednisolone was decreased to 0.5 mg/kg BID with lowered tacrolimus target of 6–10 mg/dl. Bone marrow biopsy on Day 36 showed hypocellularity (10%–20%) consistent with bone marrow involvement of GVHD. The rash fully resolved on Day 48; however, he had persistent grade 1–2 diarrhea. Initially, he was transitioned from mycophenolate mofetil to enteric-coated mycophenolate sodium but diarrhea persisted. STR analysis showed worsening donor CD3 to 84% on day 55 consistent with SR-GVHD and tacrolimus target was increased back to 8–12 mg/dl. On day 59, the patient was initiated on ruxolitinib 5 mg with a corticosteroid taper at which time liver GVHD had resolved (total bilirubin, 1.4 mg/dl). MMF was discontinued on Day 73 due to worsening thrombocytopenia (platelets, 69 × 10^9^/L). His hospital course was further complicated by Epstein–Barr virus (EBV) viremia for which he was given rituximab 375 mg/m^2^ weekly for on days 42 and 52, and he developed seizures due to tacrolimus toxicity on Day 82 for which he was transitioned to monthly belatacept after the initial loading phase.

**Figure 1:**
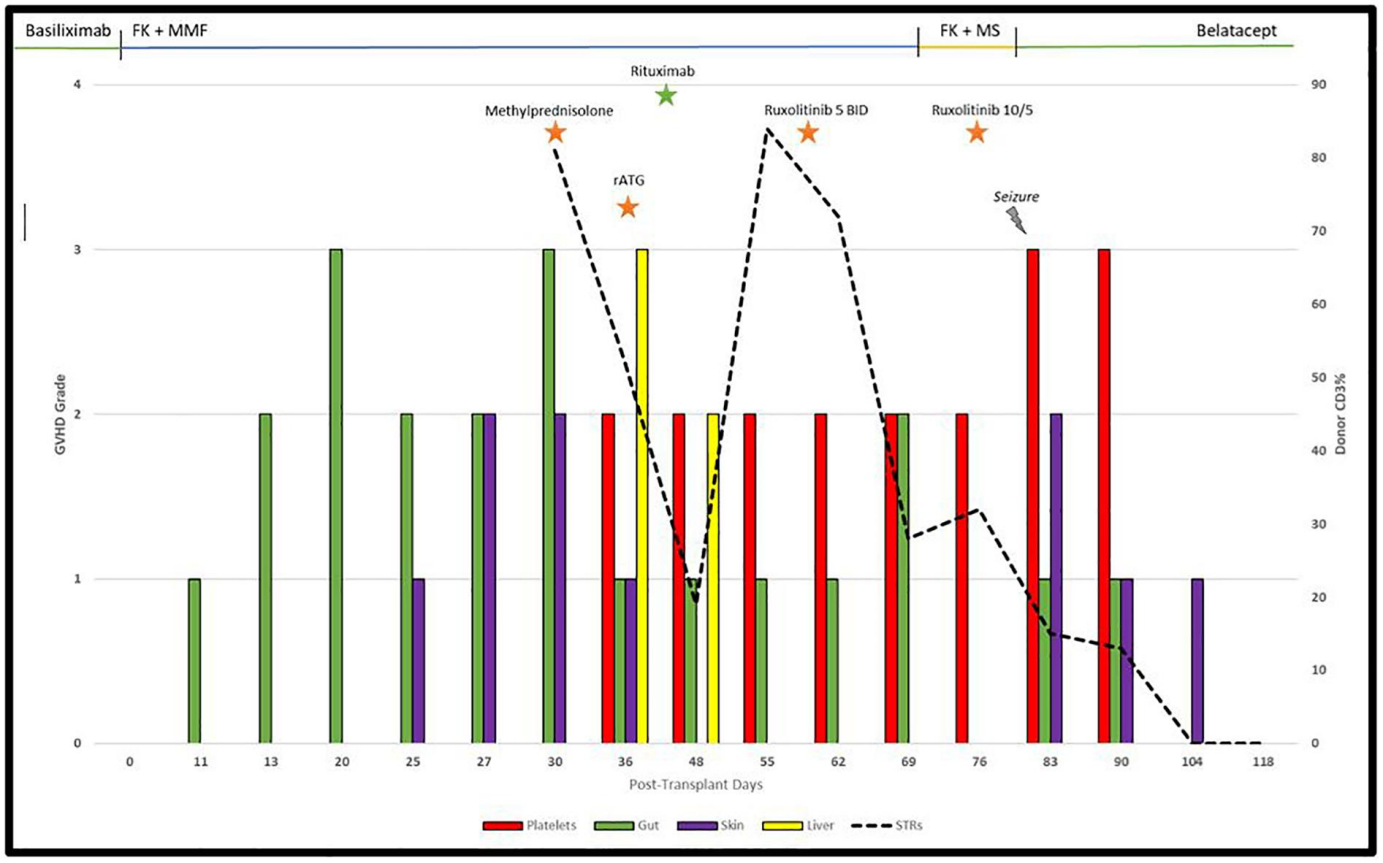
Timeline of GVHD and immunosuppression following bilateral lung transplantation.

The patient's rash worsened on Day 85 for which ruxolitinib was increased to 10 mg every morning and 5 mg every evening, and triamcinolone 0.1% cream TID was added. Platelet count normalized to 242 × 10^9^/L by Day 104. He had significant improvement in rash and diarrhea. Rash resolved completely by Day 118 at which time a ruxolitinib taper was begun. On day 203, a lung biopsy confirmed no evidence of rejection. Ruxolitinib was discontinued on Day 224. As of Day 384, there was no evidence of recurrent GVHD. The full timeline of GVHD and immunosuppression is shown in Figure 1.

## Discussion

SOT grafts contain variable amount of donor leukocytes, including monocytes, lymphocytes, NK cells and other progenitors. Typically, these cells are eliminated by the recipient's immune system and replaced with recipient lymphoid cells; however, immune tolerance to these cells while on post-transplant immunosuppression may allow the development of GVHD wherein the donor lymphocytes attack the recipient's tissues. Varying amounts of lymphocytes are present in transplanted organs and it is unknown whether the makeup of these lymphocytes or the type of transplanted organ contribute directly to the development of GVHD. Furthermore, prior studies have found risk factors for the development of GVHD following liver and intestinal transplantation, but there is limited data available to make an algorithm to predict GVHD development following lung transplantation. Our patient had 1/10 HLA matching with the donor, as HLA matching is not a standard criterion for donor selection and knowledge of the impact of HLA mismatch on GVHD following SOT is limited by its rarity. Our patient developed SR-GVHD of the skin, gut, liver and bone marrow. Diarrhea was attributed to medication side-effect in the initial hospitalization, although this likely represented the first signs of GVHD in retrospect. Although our patient's diarrhea improved initially after steroids, it was persistent and he developed liver and bone marrow GVHD prompting initiating of rATG, which was associated with significant improvement in liver enzymes and diarrhea. However, given persistent bone marrow dysfunction and rising donor chimerism, ruxolitinib was initiated for refractory GVHD, which ultimately led to complete resolution of GVHD over the subsequent two months. While data on bone marrow involvement in SOT-related GVHD and the optimal treatment approach is limited, we did initiate a donor search for HSCT as a precautionary measure. However, the patient's favorable response to IST ultimately negated the need for proceeding with HSCT.

Given the poor survival seen in patients with GVHD following SOT, it is essential to have a low threshold to obtain targeted biopsies and STR analysis if any signs or symptoms arise suggesting GVHD. Although STRs are useful as an adjunct in diagnosis of GVHD, STR testing can take a significant amount of time to return; thus, STRs should augment our primary tool in clinical monitoring.

While corticosteroids are the mainstay of GVHD treatment, it is essential to escalate immunosuppression quickly with additional agents as soon as there is evidence of worsening disease, including overlapping of multiple immunosuppressive therapies. Prior studies have shown that the majority of GVHD cases following lung transplantation are steroid-refractory (2). Given the paucity of cases, there exists no data from clinical trials to guide treatment. We suggest initiation of methylprednisolone 1–2 mg/kg immediately if evidence of GVHD while awaiting biopsy confirmation. Topical or non-absorbable oral corticosteroids can also be added if GVHD involves the skin or gut, respectively. While decreased immunosuppression may increase the host's ability to suppress donor T-cell-mediated GVHD, it also significantly increases the risk for graft rejection. Prior animal studies have demonstrated the utility of JAK inhibitors, such as ruxolitinib, in SOT graft rejection (5, 6). This is supported by our case in which the patient was on ruxolitinib, tapering steroids, and belatacept without any evidence of rejection. Accordingly, if a patient worsens or demonstrates no improvement within 3–5 days of initiating high-dose corticosteroids, we suggest adding ruxolitinib after consulting bone marrow transplant specialists for further clinical evaluation. Other treatment options for SR-GVHD may include rATG, alemtuzumab, mycophenolate mofetil, belatacept, sirolimus, extracorporeal photopheresis, or salvage allogeneic stem cell transplantation if bone marrow GVHD is present and a donor is available.

It is important to note that our patient also received two doses of rituximab for EBV viremia, which may have provided additional support in GVHD treatment as it has been used successfully to treat GVHD in several patients following bone marrow transplantation, likely through inhibition of B-cell antigen presentation to T-cells (7).

In summary, early diagnosis and treatment of GVHD is crucial as demonstrated by our case of severe SR-GVHD following bilateral lung transplantation that completely resolved with multi-agent immunosuppression. Interdisciplinary collaboration between solid organ and stem cell transplant specialists is encouraged. Further prospective studies are needed to identify the best strategies to prevent and treat GVHD following SOT.

## Data Availability

The raw data supporting the conclusions of this article will be made available by the authors, without undue reservation.
